# Zoonotic and non-zoonotic helminths in black rats of rain-fed and irrigated areas of Swat, Khyber Pakhtunkhwa, Pakistan

**DOI:** 10.1016/j.sjbs.2021.01.022

**Published:** 2021-01-28

**Authors:** Wali Khan, Noor-un-Nisa, Naseem Rafiq, Zubia Masood, Munawar Salim Ahmed, Hafeez Ur Rahman, Muhammad Kabir, Rukhsana Ghaffar, Attiqa Naz, Muhammad Ishaq Ali Shah

**Affiliations:** a Department of Zoology, University of Malakand, Lower Dir, Pakistan; b Vertebrate Pest Control Institute, Southern Zone of Agricultural Research Centre, Pakistan Agricultural Research Council, University Campus, Karachi, Pakistan; c Department of Zoology, Abdul Wali Khan University, Mardan, Pakistan; d Department of Zoology, Sardar Bahadur Khan Women University, Quetta, Pakistan; e Department of Zoology, University of Swabi, Pakistan; f Department of Zoology, Hazara University Mansehra, Pakistan; g Department of Biological Sciences, University of Sargodha, Sub-Campus Bhakkar, Bhakkar-30000, Punjab, Pakistan; h Department of Pharmacy, University of Malakand, Pakistan; i Department of Pharmacy, Abasyn University, Peshawar, Pakistan; j Department of Chemistry, Abdul Wali Khan University, Mardan, Pakistan

**Keywords:** Helminth parasites, *Rattus rattus*, Agricultural fields, Rodents, District Swat

## Abstract

Present study was conducted to get information on helminth parasites of zoonotic importance among the black rats of district Swat, Pakistan. Two hundred and sixty nine rats were captured from agricultural ecosystem of the district using live captured traps from 2011 to 2013. Captured rats were anesthetized and surveyed for the presence of ectoparasites, then were carefully dissected for investigation of endoparsites. Helminth parasites of 8 species were identified. Presence of parasite was noticed in 23.7% of sampled rats. The infection rates of sampled rats was given in order of their infectivity as *Syphacia obvelata* 13(4.83%), *Aspiculuris tetraptera* 13(4.83%), *Heterakis spumosa* 12 (4.46%), *Hymenolepis spp*. 9(3.34%), *H.diminuta* 8(2.97%), *Hymenolepis fusa* 4(1.48%), *Lutziella microacetabularae* 4(1.48%) and *Lutziella* spp. 1 (0.37%). No significant difference (P < 0.4289) was found in prevalence of parasites among areas, crops, crop stages and sex of the host while adult rats were found more infected than sub-adults. *S. obvelata* and *A. tetraptera* were the most common species of helminths while *Lutziella* sp., 1 (0.37%) was found only in one host. *Rattus rattus* (the black rat) was regarded as the host of helminth parasites of zoonotic importance, therefore the hidden health hazards of this rodent species needed to be considered to prevent infectivity of humans. Current study was concluded that *Rattus rattus* harbored a wide variety of helminth parasites which shows a hidden risk to inhabitants of the region. Monitoring rats’ population in settle areas and educating the local community about the risk of rat borne parasitic diseases transmission through rats appears to be absolutely essential.

## 1 Introduction

Rodentia is the largest order of class Mammalia and have been known to science as the most important reservoirs of parasitic infection (Etemad, 1978). Rats and mice not only act as a pest but it also acting as a prey or as a carrier/reservoir of a large number of diseases of parasitic origin (Okoye and Obiezue, 2008). Due to harboring a large number of zoonotic importance rats showing threats to human health who live in close vicinity to rodent populations (Zain et al., 2012). External parasites like mites, lice and ticks can transmit a number of pathogens to man and their animals (Soliman et al., 2001). The eggs of helminth parasites are passed out in rodent droppings on agricultural products, stored grains and in various edible items in houses and thus responsible for spreading of the disease (Khatoon et al., 2004). Their ability to act as a vector is greatly enhanced due to their physiological similarities which they share with humans (Kataranovski et al., 2010). Hence increased rodent population in an area could be directly related to increased zoonotic diseases in human population (Stojcevic et al., 2004). Zoonotic importance of rodents had also been attracted the attention of WHO experts (WHO, 2007).

The helminth parasites of rats have been studied in many parts of the world with special emphasis on zoonotic parasites. In Pakistan *R. rattus, R. norvegicus* and *R.r. rufescens* have been trapped from different regions of Pakistan and screened for helminth parasites. *Protospirura muris* in nematodes and *H. nana* (fraterna) in cestodes were the most prevalent helminths isolated from *R. rattus*, however, trematodes and acanthocephalans were the least groups of parasites reported from *R. rattus* in Pakistan.

Published data on distribution of rats and their parasites is rare in Pakistan and need special consideration to be studied. There are some reports on the endoparasite infestation of *R. rattus* in Pakistan such as: Lahore, Pakistan (Akhtar, 1955, Ahmad et al., 2014). Endoparasites have also been isolated from *R. rattus* in Karachi, Pakistan (Fahim, 1960, Bilqees and Siddiqui, 1981, Farooq and Yousuf, 1986, Mehr run Nisa and Shimi, 1986). Helminth parasites were also have been detected in *R. rattus* in Rawalpindi-Islamabad (Faiz-ul-haque et al., 1990); Faisalabad (Rafique et al., 2009) and Swat district, Pakistan (Noor-un-Nisa et al., 2013).

Keeping in view that rat born parasites have not been studied previously from district Swat, Pakistan. Current study was therefore aimed to gather information about the parasites of *R. rattus* in agricultural habitats, where they are in close association with human settlements, with focus on parasites, which have medical importance for implementation of any prevention and control measures in Swat region particularly and in Pakistan generally.

## 2 Materials and methods

### 2.1 Study area

Current research was carried out from 2011 to 2013 in Swat, district Pakistan, situated with 34°34° to 35° 55° North and 72° 08° to 72° 50° East to determine the prevalence of zoonotic and non-zoonotic parasites in *R. rattus* of agricultural pests. Swat is located 247 km north of the capital Islamabad at an altitude of 984 m above sea level (Khan et al., 2018) . In winter season temperature decreases to −2 °C and in summer it increases to 33 °C. Agriculture is the main occupation followed by live-stock.

### 2.2 Rats trapping and identification

Live-capture traps were positioned near tunnels and the ways of rats’ entrance in each of the agricultural fields. The oily bread was used as baits in the traps. All the traps were set before sunset and were collected next early in the mornings. Some of the rats (as voucher specimens) were transported to vertebrate Pest Control Institute, Pakistan Agricultural Research Centre, University Campus, Karachi, Pakistan for confirmation of species identification.

### 2.3 Isolation of parasitic worms

Each of the rat specimens was anaesthetized and weighted through Precisa balance model No.18220 Switzerland. Each of the rats was identified up to species level and the information concerned was gathered relating the sex and age by using valid identification keys (Aplin et al., 2003). Chloroform was used to anesthetize and killed the rats in a vacuum chamber. Each specimens of the rats were dissected. During dissection, their digestive tracts were detached and the contents of each part were diagnosed. The lining membrane of intestines was scraped with a sharp blade and the contents were studied under a stereomicroscope. Cestodes and trematodes were stained with carmine acid while the nematodes were placed in lactophenol for identification on morphological characterization (Yamaguti, 1958).

### 2.4 Ethical statement xxx

Collection and processing of the rat specimens was performed in accordance with the procedures approved by the Ethical Committee of Pakistan Agricultural Council Islamabad and University of Karachi.

### 2.5 Identification and mathematical calculation of helminth parasites

Collected helminth parasites were identified by Yamaguti’s method (Yamaguti, 1954). Incidence, intensity, density, relative density and index of infection were calculated by applying the formula of (Gaherwal et al., 2011).

## 3 Results

Two hundred and sixty nine rats were caught. Eight species of helminthes were recorded from the gastrointestinal tract contents of the rats. Sixty four specimens of the reported helminthes were observed, resulting in a prevalence of 23.7% with single or multiple infection.

### 3.1 Parasite infestation of sampled rats according to the region

Two hundred and sixty nine rats (*R. rattus*) were caught in this study. Of the total 23.7% (n = 64/269) were found infected. Slightly higher prevalence rate 12.6% (34/269) was found in the rats of irrigated areas than in the rain-fed 11.1 (n = 30/269) of sampled rats. No statistical difference (P-value = 0.9190) was noted between the areas and that of parasites recorded (Table 1).

**Table 1:** Prevalence of helminth parasitic infection in rats of district Swat, Pakistan.

Parasite species	areas		Total (%)	Chisquare test	P-value
	Rain-fed	Irrigated		2.604	0.9190
*S. obvelata*	7	6	13(4.83)		
*A. tetraptera*	7	6	13(4.83)		
*H. spumosa*	5	7	12(4.46)		
*Lutziella* sp.	0	1	1(0.37)		
*L. microacetabularae*	1	3	4(1.48)		
*H. fusa*	2	2	4(1.48)		
*Hymenolepis* sp.	4	5	9(3.34)		
*H. diminuta*	4	3	7(2.60)		
Total infected	30(11.1)	34(12.6)	64(23.7)		
Total examined	128(47.5)	141(52.4)	269		

### 3.2 Parasite infestation of sampled rats according to the crops

Highest prevalence rate 7.06% (19/269) was investigated in the rats of rice fields followed by maize rain-fed 631% (n = 17/269) while the least infection was observed in the rats caught in potatoes 4.83% (n = 13/269). No statistical difference (P-value = 0.6912) was noted between the crops and that of parasites recorded (Table 2).

**Table 2:** Prevalence of helminth parasitic infection in rats of different crops of district Swat, Pakistan.

	Crops				Total (%)	P Value
Parasite species	Potatoes	Maize (R)	Maize (I)	Rice		0.6912
*S. obvelata*	3	4	4	2	13(4.83)	
*A. tetraptera*	3	4	4	2	13(4.83)	
*H. spumosa*	2	3	3	4	12(4.46)	
*Lutziella* sp.	0	0	0	1	1(0.37)	
*L. microacetabularae*	0	1	2	1	4(1.48)	
*H. fusa*	1	1	2	0	4(1.48)	
*Hymenolepis* sp.	2	2	2	3	9(3.34)	
*H. diminuta*	2	2	2	2	7(2.60)	
Total infected	13(4.83)	17(6.31)	15(5.57)	19(7.06)	64(23.7)	
Total examined	53	75	66	75		

### 3.3 Parasite infestation of sampled rats’ season wise

The rats captured during mature/harvesting period were found more infected 9.29% (n = 25/269) followed by flowering/fruiting stage 8.55% (n = 23/269) while the least 5.94% (n = 16/269) rate of infection was found in the rats caught at vegetative stage of the crops studied. No statistical difference (P-value = 0.4141) was noted between the crop stages and that of parasites recorded (Table 3).

**Table 3:** Prevalence of helminth parasitic infection in rats of different seasons in district Swat, Pakistan.

	Seasons			Total (%)	P Value
Parasite species	Vegetative	Flowering/fruiting	Mature/harvesting		
*S. obvelata*	4	5	4	13(4.83)	0.4141
*A. tetraptera*	3	6	4	13(4.83)	
*H. spumosa*	3	3	6	12(4.46)	
*Lutziella* sp.	0	1	0	1(0.37)	
*L. microacetabularae*	1	2	1	4(1.48)	
*H. fusa*	0	1	3	4(1.48)	
*Hymenolepis* sp.	3	2	4	9(3.34)	
*H. diminuta*	2	3	3	7(2.60)	
Total infected	16(5.94)	23(8.55)	25(9.29)	64(23.7)	
Total examined	60	104	105		

### 3.4 Parasite infestation of sampled rats sex wise

Of the sampled rats males were more infected 15.9% (n = 43/269) than females 7.80% (n = 21/269). No statistical difference (P-value = 0.7914) was noted between the sex of the rats and that of parasites recorded (Table 4).

**Table 4:** Prevalence of helminth parasitic infection in rats of both sex in district Swat, Pakistan.

	Sex		Total (%)	Chi-square test	
Parasite species	Male	Female		Chi-square	P-value
*S. obvelata*	9	4	13(4.83)	3.898	0.7914
*A. tetraptera*	6	7	13(4.83)		
*H. spumosa*	9	3	12(4.46)		
*Lutziella* sp.	1	0	1(0.37)		
*L. microacetabularae*	3	1	4(1.48)		
*H. fusa*	3	1	4(1.48)		
*Hymenolepis* sp.	6	3	9(3.34)		
*H. diminuta*	6	2	7(2.60)		
Total infected	43(15.9)	21(7.80)	64(23.7)		
Total examined	165	104			

### 3.5 Parasite infestation of sampled rats age wise

Adult rats were more infected 20.0% (n = 54/269) than sub-adults 3.71% (n = 10/269). No statistical difference (P-value = 0.6852) was noted between the age of the rats and that of parasites recorded (Table 5).

**Table 5:** Prevalence of helminth parasitic infection in sub-adults and adults in district Swat, Pakistan.

	age		Total (%)	Chis-square test	
Parasite species	Sub-adults	adults		Chi-square	P-value
*S. obvelata*	3	10	13(4.83)	4.793	0.6852
*A. tetraptera*	2	11	13(4.83)		
*H. spumosa*	3	9	12(4.46)		
*Lutziella* sp.	0	1	1(0.37)		
*L. microacetabularae*	0	4	4(1.48)		
*H. fusa*	0	4	4(1.48)		
*Hymenolepis* sp.	2	7	9(3.34)		
*H. diminuta*	0	8	7(2.60)		
Total infected	10(3.71)	54(20.0)	64(23.7)		
Total examined					

### 3.6 Evidence on zoonotic parasites

A total of 23.7% (64/269) of the sampled population was infected with helminth parasites, of them 4.46% (12/269) have been identified as zoonotic parasites. Detected helminth parasites of zoonotic importance comprised 2 species such as: *H. nana* 4/269 (1.48% and *H. diminuta* 8/269 (2.97%).

### 3.7 Non-zoonotic parasites

Totally, 19.3% (52/269) were detected as non-zoonotic species. *Hymenolepis* sp. 9/269 (3.34%); *S. obvelata* and *H. spumosa* 13/269 (4.83% each); *A. tetraptera* 12/269 (4.46%); *L. microacetabularae* 4/269 (1.48%) and *Lutziella* spp. 1/269 (0.37%) species of helminths with no medical importance were observed during microscopic examination of the fecal wet and stained smears (Table. 6). Eggs were differentiated based on egg morphology as described by Baker (2007) Fig. 1.

**Table 6:** Detected helminth parasites of zoonotic and non-zoonotic importance in the sampled rats of Swat, Pakistan.

Zoonotic	Rats examined	Infested rats	Infection (%)
*H. nana*	269	4	1.48
*H. diminuta*	269	8	2.97
Sub-total	–	12	4.46
Non– Zoonotic			
*Hymenolepis* sp.	269	9	3.34
*S. obvelata*	269	13	4.83
*A. tetraptera*	269	12	4.46
*H. spumosa*	269	13	4.83
*L. microacetabularea*	269	4	1.48
*Lutziella* sp.	269	1	0.37
Sub-total	–	52	19.3
Gross total	269	64	23.7

**Fig. 1:**
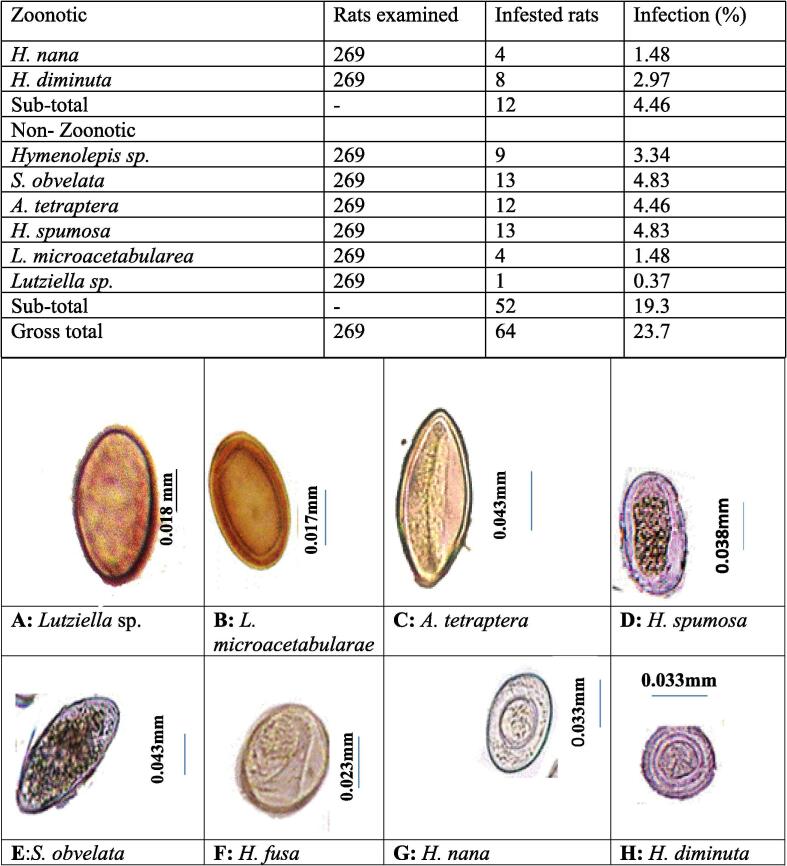
Photomicrographs of helminth eggs. Identification of each helminth egg is given along with its average size, length (mµ).

## 4 Discussion

Studies on intestinal helminth parasites from *R. rattus* are scarce in Pakistan. In general, 23.7% of the rats were infected with at least one species of helminth parasites. Seeing the variation of parasites, eight species of helminths were found in *R. rattus* presently with *A. tetraptera, S. obvelata, H. spumosa, H. diminuta, H. nana, H. fusa, L. microacetabularae* and *Lutziella* sp. Findings of the current study were similar to some extent with those of previous studies in Pakistan.

The highest prevalence of infection with helminth parasites was seen in males, although no significant difference was seen between male and female rats (*P* > 0.05). This might be owing to more activity or higher number of the males. The highest parasite infestation rate was detected in sampled rats of adults (20%) than in sub-adults (3.71%). Higher parasite infestation rate in adults can be due to higher activity in the habitat. Rats trapped in the harvesting stage of the crops were most infected (9.29%) than others. Crop wise rats from potatoes fields were slightly more infected (7.06%) as compared to others and 12.6% highest infection rat was noted in the rats captured in the irrigated areas.

Several researchers have isolated *H. diminuta, H. nana* and *H. fusa* from *R. rattus* in different parts of Pakistan (Fahim, 1960; Henry et al., 1971; Bilqees and Siddiqui, 1981, Faiz-Ul-Haque et al., 1990, Rafique et al., 2009, Ahmad et al., 2014). In this study*, A. tetraptera, S. obvelata, H. spumosa* and *Lutziella* spp. were seen in *R. rattus* for the first time in Pakistan. Lutziella microacetabularae was identified in *R. rattus* of Swat, Pakistan this (Noor-un-Nisa et al., 2013), however, in present study, this parasite was also determined.

The species of the genus *Rattus*, such as *R. rattus, R. rattus rufescens* and *R. norvegicus* have observed in Pakistan. The helminth parasites found in the above listed rats species, with the exception of *A. tetraptera, S. obvelata* and *H. spumosa,* for the first time in Pakistan (table 6). However, this rat had have a number of parasite species in Pakistan such as: *A. lahorica* (Akhtar, 1955); *Protospirura muris, Syphacia* sp., *R. railietina celebensis, H. murinis, Cysticercus* larvae (Fahim, 1960); *A. pakistanica, S. muris, T. muris, Gongylonema neoplasticum, P. muris, Rictularia* spp. *Mathevotaenia symmetrica, T. taeniformis, Monttiformis dubins* (Henry et al., 1971); *Moniliformis karachinensis* n.sp. *Acanthocephalis murinis* n.sp. and *Strobiocercus of Taenia taeniformis* (Bilqees and Siddiqui, 1981); *Euparyphium lobata* sp.n (Mehr run Nisa and Shimi, 1986); *T. taeniformis* and *Trichuris* sp. (Rafique et al., 2009); *Lutztrema* (*Lutziella*) *microacetabularae* Rohde, 1966 (Noor-un-Nisa et al., 2013).

In present study only two species such as *Hymenolepis nana* (1.48%) and that of *H. diminuta* (2.97%) were reported zoonotically important. In a study conducted in Kashan, the zoonotic parasites *H. nana fraterna* (10.8%), *H. diminuta* (4.2%), and *Trichuris muris* (1.7%) were identified in 120 rodents including *Meriones persicus*, *M. libycus*, *Gerbillus nanus*, *M. musculus*, *R. norvegicus*, *R. rattus* and *Jaculus blanfordi* (Rasti et al., 1998).

In a research on 90 rodents including *R. norvegicus*, *R. rattus*, and *M. musculus* in Ahvaz, the parasites *Trypanosoma lewisi*, *Trichosomoides crassicauda*, *Gongylonema monigi*, *Streptopharagus kuntzi*, and *Rictularia ratti* were identified; none of these parasites resembled the ones found in the present study (Kia et al., 2001). This might be due to the difference in geographical locations and climatic conditions.

The most commonly recognized zoonotic parasite in the present study was *H. diminuta* (the rat tapeworm) with the highest infestation rate in *R. rattus*. This is consistent with the results of (Fahim, 1960) in Karachi; (Faiz-ul-haque et al., 1990). *H. nana* was reported 1.48% in prevalence in the present study. This parasite was reported in different murid in Pakistan as Rawalpindi-Islamabad (Faiz-ul-haque et al., 1990); Faisalabad (Rafique et al., 2009). Current study findings were comparable with studies conducted by (Kia et al., 2010) with 11.1% infection rate of *H. diminuta*. It has been observed that prevalence rate of infection with *H. diminuta* is not similar in rats of at different localities.

## 5 Conclusions

Considering of rodents’ parasitic fauna in diverse regions of the world can bridge the gap of information about the possible potentials for transmission of zoonotic helminths to humans in the region. North-western Pakistan is a part with unanswered questions upon this issue. The study of parasitic fauna in rodents in North-Western of the country is an attracting subject to parasitologists interested to recognize the role of rodents in zoonotic infection transmission. The results suggested that the range of various parasites recovered from the fecal droppings of rats collected from agricultural habitats can make human habitation more vulnerable to parasitic zoonosis and may thus be of significant public health importance. Further studies are required to understand the cross species transmission among the rats and human beings.

## Declaration of Competing Interest

The authors declare that they have no known competing financial interests or personal relationships that could have appeared to influence the work reported in this paper.
